# Progress of tissue adhesives based on proteins and synthetic polymers

**DOI:** 10.1186/s40824-023-00397-4

**Published:** 2023-06-07

**Authors:** Gi-Yeon Han, Soo-Kyung Hwang, Ki-Hyun Cho, Hyun-Joong Kim, Chong-Su Cho

**Affiliations:** 1 Program in Environmental Materials Science, Department of Agriculture, Forestry and Bioresources, Seoul National University, Seoul, 08826 Korea; 2 Research Institute of Agriculture and Life Sciences, Seoul National University, Seoul, 08826 Korea; 3 Department of Plastic and Reconstructive Surgery, Seoul National University Hospital, Seoul, 03080 Korea

**Keywords:** Tissue adhesive, Natural polymer, Synthetic polymer, Next-generation

## Abstract

In recent years, polymer-based tissue adhesives (TAs) have been developed as an alternative to sutures to close and seal incisions or wounds owing to their ease of use, rapid application time, low cost, and minimal tissue damage. Although significant research is being conducted to develop new TAs with improved performances using different strategies, the applications of TAs are limited by several factors, such as weak adhesion strength and poor mechanical properties. Therefore, the next-generation advanced TAs with biomimetic and multifunctional properties should be developed. Herein, we review the requirements, adhesive performances, characteristics, adhesive mechanisms, applications, commercial products, and advantages and disadvantages of proteins- and synthetic polymer-based TAs. Furthermore, future perspectives in the field of TA-based research have been discussed.

## Introduction

The use of tissue adhesives (TAs) as a sutureless technique is receiving interest in surgical applications owing to several advantages, such as less surgery time, infection mitigation, leakage prevention, easy application, less pain, no requirement for subsequent removal procedures, and minimal-invasive surgery to restore functionality and soft tissue integrity [1]. Although suturing is regarded as the gold standard for reconnecting surgical incisions owing to its simple procedure, several limitations, such as the time-consuming procedure, anesthesia requirement, possibility of infection, granuloma formation, and need for various surgical skills [2], restrict its applicability and compel the development of sutureless TAs. An ideal TA should exhibit certain properties, such as:

1. biocompatibility with non-local irritation, anti-inflammatory activity, non-toxicity, and non-antigenicity [1];
2. easy applicability on the target tissue surface;
3. biodegradability after exerting their functions;
4. the occurrence of the reticulation process in the presence of body fluids in a short time, based on the operation requirements [1];
5. pliability similar to the target tissue to follow expansion/contraction based on the physiological conditions of the target tissue [1];
6. a strong binding efficacy to ensure adequate mechanical properties;
7. the maintenance of bonding in a wet physiological environment [3].

However, the existing TAs can meet only a few of the aforementioned requirements. The selection of an appropriate TA for a specific application depends on the required properties based on the specific target tissue.

Polymeric TAs can perform a wide range of functions because of the generation of a three-dimensional network that binds to the target tissue [1]. These TAs are composed of natural or synthetic polymers. Natural polymeric TAs are protein-based TAs, such as those formed using fibrin, gelatin, and albumin, with high biocompatibility and low adhesive strength. Synthetic polymeric TAs, including polycyanoacrylate (PCA), poly(ethylene glycol) (PEG), and polyurethane (PU), exhibit high adhesive strength and low biocompatibility. Both types of polymeric TAs can be applied to tissues.

Herein, we have reviewed the requirements of polymeric TAs and principles of adhesives as well as the characteristics, adhesive mechanisms, applications, commercial products, and advantages and disadvantages of proteins- and synthetic polymer-based TAs. Additionally, future research directions have been discussed.

## Principles of adhesives

### Characteristics of adhesives

Adhesives can hold materials together by interactions between interfacial of materials such as mechanical interlocking, Van der Waals force, and hydrogen bond. The interactions occurred between adhesives and adherend is can divide into chemical and physical interactions [4]. Types of adhesives can be categorized into general, hot-melt, and pressure-sensitive adhesives (PSAs) based on the contact and curing process. General adhesives, such as polyepoxy adhesives and PCA, adhere to a liquid state and subsequently to a solid state through an irreversible curing process. Hot-melt adhesives are applied in the melted state by heating, and adhesion is achieved via a phase transition (liquid to solid). PSAs are adhesives that maintain their stickiness at room temperature and adhere without additional reactions or phase transitions. Each type of adhesive has different applications based on its functional properties. Polyepoxy adhesives, which are a type of general adhesives, are widely used as structural adhesives owing to their good mechanical properties and high adhesion strength when used between dissimilar materials [5]. Hot-melt adhesives are commonly used in the packaging industry because of their ease of application [6]. PSAs are used in the biomedical and display fields owing to their flexibility [7].

### Adhesion strength

Adhesion strength is a measured force when external forces (e.g., gravity and peeling) are applied to the adhesives for separating two objects. The adhesion strength is represented by the work of adhesion (ω) as shown in Eq. 1 [8]:

1 $$\mathrm\omega\;=\;{\mathrm\gamma}_{\mathrm{adhesive}}+{\mathrm\gamma}_{\mathrm{substrate}}-{\mathrm\gamma}_{\mathrm{interface}}$$

Where γ represents the surface energy.

The work of adhesion is governed by the physical and chemical properties of adhesives and substrates [9]. The interactions are categorized into physical interactions, such as interpenetration and interlocking [10, 11], and chemical interactions such as covalent bonds, hydrogen bonds, and electrostatic interactions [12, 13]. The stronger the interaction, the stronger the adhesion. In addition, the viscosity and strength of the adhesives are important factors. Adhesives with high viscosity, do not sufficiently wet the substrate. Furthermore, adhesives with a low modulus, do not exhibit sufficient adhesive strength and are destroyed when an external force is applied. These factors and interactions between the adhesives and substrates collectively affect the adhesion strength.

### Methods to measure the adhesion strength

**Fig. 1:**
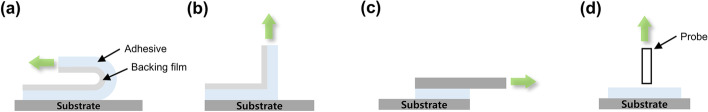
Schematic of adhesion testing methods: **A** 180° peel test, **B** 90° peel test, **C** lap shear test, and **D** probe tack test

**Fig. 2:**
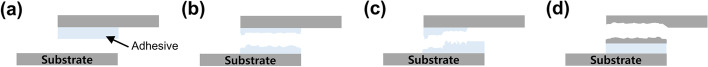
Schematic of adhesion failure modes: **A** interfacial failure, **B** cohesion failure, **C** mixed failure, and **D** substrate failure

Adhesion strength is a combination of adhesion (bonding strength) forces and cohesion (strength of adhesive itself) of adhesives. Peel and lap shear tests are commonly used to evaluate adhesion strength. During the peel test, an adhesive is attached to a substrate, and subsequently, it is peeled off in a direction parallel (180°) (Fig. 1A) or perpendicular (90°) (Fig. 1B) to the substrate. The peel test measures the force required to remove the adhesive from the substrate. The lap shear test (Fig. 1C) measures the force required to detach the adhesive by applying a force parallel (shear direction) to the interface when the adhesive is attached between the substrates. Furthermore, the initial contact adhesion is an important factor, particularly in PSAs, and is measured using a probe tack (Fig. 1D). The probe tack measures the force required to remove the probe (commonly a cylindrical probe) that is in contact with the adhesive in a direction perpendicular to the adhesive. Additionally, the failure mode must be measured during the adhesion test. The failure mode can be categorized into as followed: 1) interfacial failure (Fig. 2A) when destruction occurs between the adhesive and interface because of a lack of adhesion, 2) cohesion failure (Fig. 2B) when the adhesive is destroyed because of a lack of cohesion, 3) mixed failure (Fig. 2C) when the interfacial and cohesion failure occurs together, 4) substrate failure (Fig. 2D) when the substrate is destroyed because of excessive adhesion. The degree of interaction between adhesive and substrate and adhesive strength is determined by evaluating the failure mode.

### Wetting and underwater adhesion

An adhesive must be sufficiently wetted on the adherend to facilitate good adhesion [9]. Good wetting increases the specific surface area where adhesive and substrate can interact, however, a water layer is formed between the substrate and adhesive in an underwater and moist environment [14]. Water is a polar molecule that can form hydrogen bonds with other water molecules [8]. Therefore, they interfere with the hydrogen bonding between the adhesive and substrate. Water prevents direct interactions between the adhesive and substrate, thereby lowering the adhesion strength. The addition of a filler to absorb water [15] or the introduction of hydrophobic moiety in the polymer network [16] are common methods to repel water. Recently, hydrophilic polymers have been applied in a dry state to absorb water [17]. And by forming microstructures on the surface of the adhesive to drain water [18].

### Hydrogel adhesives

Hydrogels have received considerable attention in the biomedical field owing to their high water content and similarity to the extracellular matrix [19]. Moreover, they can be used as functional materials by adding additives such as conductive molecules and drugs [20, 21]. The use of hydrogel adhesives has received significant attention, particularly as an alternative to conventional wound closure methods, such as suturing and stapling [22]. The adhesion performance of hydrogel is a crucial factor in wound dressing and significantly affects the durability of the hydrogel. However, the hydrogel matrix comprises water, therefore, obtaining an adequate adhesion is difficult. The work of adhesion is different in a moist environment compared to that in a dry environment shown in Eq. (2) and (3) [8]:

2 $$\mathrm\omega={\mathrm\gamma}_{\mathrm{hydrogel}}+{\mathrm\gamma}_{\mathrm{substrate}}-{\mathrm\gamma}_{\mathrm{interface}}$$

3 $${\mathrm\gamma}_{\mathrm{hydrogel}}\;=\;{\mathrm\Phi}_{\mathrm s}\;\;{\mathrm\gamma}_{\mathrm{network}}\;+\;\left(1\;-\;\mathrm\Phi\right)\;{\mathrm\gamma}_{\mathrm{water}}$$

where Ф_s_ represents the polymer content of the gel. The surface energy of hydrogel is similar to the surface energy of water water (i.e., γ_hydrogel_≒ γ_water_) when a significant amount of water (Ф_s_ ≒ 0) is present in the hydrogel matrix.

### Tissue adhesion

Tissue adhesives require more consideration than the adhesives used for general substrates, such as stainless steel, glass, and aluminum because: 1) the human skin is a layer comprising of proteins; therefore, several polar groups, such as carboxylic acids, hydroxyl groups, and thiols are present in the skin [23], 2) the skin is rich in moisture, therefore, the surface exists as a moist environment. Moreover, the pH sensitivity of the adhesive should be considered depending on the application area [13]. For example, sweat from the skin and wound site exhibits low acidity, whereas the organs in the stomach are highly acidic. Body fluids and blood are slightly basic. If the pH of the application area and the pH sensitivity of the materials are not considered, the adhesives may not function adequately or their rapid degradation may occur. For example, a hydrogel can absorb a significant amount of water and expand, causing pressure on surrounding tissues.

## Representative examples of polymeric TAs

### Natural polymer-based TAs

#### Fibrin

##### Characteristics

Fibrin adhesives have been used for more than 100 years, thus they have the longest history among all adhesives [24]. Fibrin was first applied to human patients in 1924 [25] and was approved by the FDA of the United States in 1998 [22]. The fibrin TAs comprise several substances, such as fibrinogen, thrombin, factor XIII, and Ca^2+^ ions [22]. They are normally derived in the body; therefore, they exhibit biocompatibility, biodegradability, non-inflammatory and non-foreign body reactions, and non-tissue necrosis [22]. The clinically approved fibrin TAs are listed in Table 1.

**Table 1:** List of clinically approved fibrin adhesives (modified from ref. [22])

Company name (country name)	Trade name	Components	Clinic applications	Ref
**Baxter(USA)**	Tisseel	Human fibrinogen, thrombin, fibronection bovine aprotinin	Hemostasis in surgery	[26]
**Baxter(USA)**	Artiss	Gelatin granules and human thrombin	Hemostasis	[27]
**Ethicon, J & J(USA)**	Evarrest	Human fibrinogen, thrombin	Hemostasis	[27]
**Ethicon, J & J(USA)**	VISTASEAL	Human fibrinogen, thrombin	Hemostasis in surgery	[28]
**Haemacure(Canada)**	Hemaseel	Human fibrinogen, thrombin, bovine thrombin	Hemostasis	[27]
**Ethicon, J & J(USA)**	Evicel	Human fibrinogen, thrombin, fibronectin	Hemostasis on the liver surface	[27]
**Stryker(USA)**	Vitagel	Bovine collagen, bovine thrombin, patient's own plasma	Hemostasis in surgery procedure	[29]
**Baxter(USA)**	Tachosil	Equine collagen patch, human fibrinogen, human thrombin	Hemostasis in cardiovascular and hepatic forgery	[30]
**LFB-Lille(France)**	Biocol	Human fibrinogen, thrombin, polyphosphate, calcuim	Hemostasis (Commercially approved TA-2)	[31]
**KM Biologics Co., Ltd. (Japan)**	Bolheal	Human fibrinogen, thrombin, coagulation factor XIII, calcium	Hemostasis	[31]
**Aventis Behring** **(Germany)**	Beriplast P	Human fibrinogen, thrombin, coagulation factor XIII, calcium	Hemostasis	[31]

##### Mechanism of adhesion

**Fig. 3:**
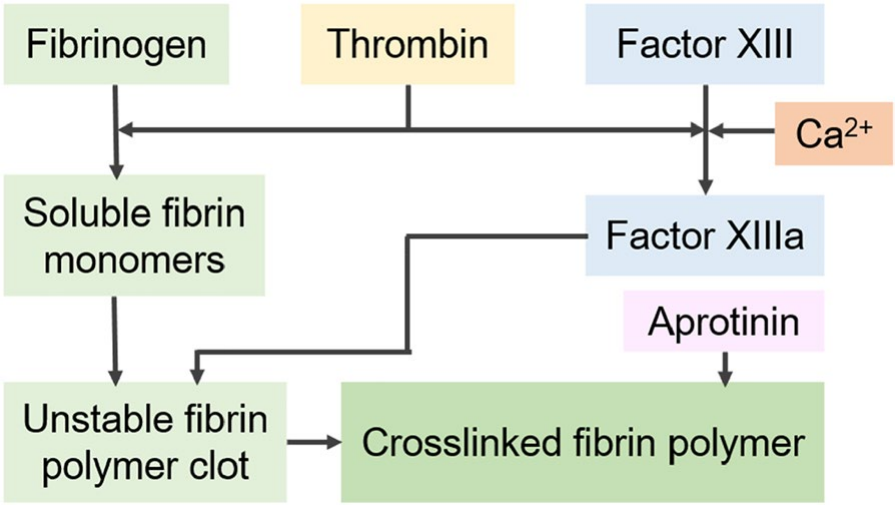
Schema of fibrinogen and thrombin interaction to yield a mature fibrin glue clot (Adapted from Bao et al. Materials Science & Engineering C. 2020;111:110,795 with permission from Elsevier [32])

For fibrin adhesion, a two-component solution, comprising fibrinogen with factor XIII and thrombin with Ca^2+^, is reconstituted in two separate syringes. The thrombin cleaves peptides A and B of fibrinogen during mixing to form a fibrin monomer. Subsequently, the fibrin monomers self-assemble into an unstable fibrin polymer network via hydrogen bonding. Finally, factor XIII, activated by the thrombin, catalyzes the chemical crosslinking via amide bonding as shown in Fig. 3 [32].

#### Applications

##### Hemostatic application

Severe hemorrhages induce significant metabolic and cellular dysfunction and may cause death in the case of continuous bleeding. Fibrin was first reported as a hemostatic agent in 1909 [26]. Since then, the fibrin TAs are widely used in different surgeries, such as cardiovascular, hepatic, pancreatic, thoracic, lung, endoscopic sinus, and total knee arthroplasty surgeries [27]. Additionally, they are used in fistula closure and traumatic abdominal injuries [29, 33]. Furthermore, currently, bleeding peptic ulcers are treated using noninvasive endoscopic injections instead of surgical procedures [34]. Sundaram et al., prepared in situ gel forming TAs with tigecycline(TI)-loaded GE NPs consisting of fibrin and chitin for controlling bleeding and preventing bacterial infection at mediastinum [35] because the chitin promotes hemostasis [36] and slows the rate of fibrin degradation, and the TI has antibacterial properties [37]. The TAs exhibited in situ gel formation within a minute with an excellent TA property when the fibrin part and chitin one were injected together. Also, the TAs showed rapid blood clotting potential by an achievement of hemostasis within 84 s under liver bleeding conditions of rats, indication of controlling bleeding and prevention of bacterial infection after cardiac surgery.

##### Orthopedic application

**Fig. 4:**
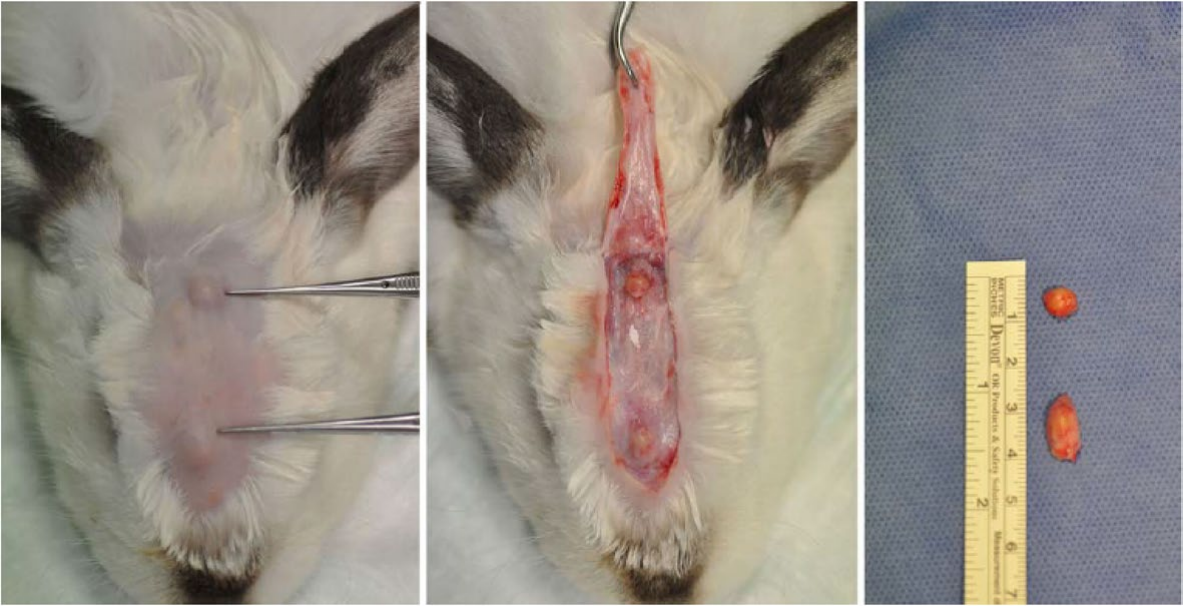
The new tissue formations in the forehead and interocular regions of a rabbit at 8 weeks after the implantation (left), during dissection (middle), and after resection (right). The ruler measures centimeters. (Adapted from Cakmak et al. The Laryngoscope. 2013;123:2986–2992 with permission from The American Laryngological Rhinological and Otological Society, Inc [40])

Fibrin TAs are used for successful orthopedic treatment, including pain reduction, symptom improvement, and long-term functionality [38]. The fibrin monomer can be polymerized into chondrocyte-loaded moldable hydrogels by thrombin; therefore, it was used to encapsulate chondrocytes for preparing tissue-engineered neocartilage [39]. The chondrocyte-loaded fibrin glue exhibited high-quality neocartilage after 12 weeks of implantation of chondrocyte-loaded gels in mice. Fibrin glue and platelet-rich (PR) fibrin glue were used to load bone marrow-derived human mesenchymal stem cells (BM-hMSCs) for developing articular tissue-engineered cartilage [38]. The loading of BM-hMSCs in fibrin glue and PR-fibrin glue increased the expression of the collagen II gene after 2.5 weeks of the implantation of the BM-hMSC-loaded gels, although no difference was observed between fibrin glue and PT-fibrin glue. Cakmak et al. [40] loaded chondrocytes in fibrin glue for preparing injectable tissue-engineered cartilage. The loading of chondrocytes in the fibrin glue led to the formation of new tissues in round, elliptical, and flat shapes after 8 weeks of injection into the forehead and interocular regions of the rabbits (Fig. 4).

Total hip arthroplasty (THA) is the major orthopedic surgery for curing end-age osteonecrosis of the femoral head, traumatic conditions, and degenerative arthritis [41]. Fibrin glue is used in THA to reduce postoperative blood loss and transfusion [42] although the fibrin glue itself will be resorbed by the physiological process of fibrinolysis induced by proteolytic cleavage and activation of plasminogen to plasmin [43]. Therefore, many researches have been tried for prevention of various antifibrinolytic agents. Kearns et al. [42] compared the reduction in total blood loss during THA using fibrin glue containing aprotinin as an antifibrinolytic agent of TISSEL with that using intravenous (IV) tranexamic acid (TXA). The minimum postoperative hemoglobin level was significantly lower in TISSEL patients with a reduction in total blood loss than that in IV TXA patients. Mahmood et al. [44] compared the reduction in blood loss using fibrin glue with that using IV TXA during total hip replacement (THR) in patients. Although IV THA performed better than fibrin glue in reducing the postoperative transfusion requirements, no significant difference was observed in wound leakage. Nasal septal cartilage is the primary material used for nasal reconstruction because of its ease of harvest, minimal donor site morbidity, and mechanical properties for nasal support; however, autologous cartilage has certain limitations [45]. Fibrin glue is used for tissue-engineered cartilage; however, its use is restricted owing to fast degradation and weak mechanical properties [46]. Gupta et al. [45] used fibrin-genipin hydrogel instead of using fibrin alone for tissue-engineered cartilage in nasal reconstruction to overcome the aforementioned limitations; however, the hydrogel formulations exhibited lower modulus of the tissue-engineered cartilage than that of the rabbit nasal septal cartilage.

##### Advantages and disadvantages

Fibrin TAs offer several advantages, such as supporting cell growth, non-toxicity, biocompatibility without inflammatory responses, foreign-body reactions, tissue necrosis, and fibrosis, and biodegradability, because they are derived from components found in the human body [29]. However, they also have several disadvantages, such as poor mechanical properties with a low cohesion strength, the transmission of blood-borne disease due to the virus, and the risk of allergic or anaphylactic reactions when bovine-derived substances are used [30].

#### Gelatin

##### Characteristics

Gelatin derived from the thermal denaturation of collagen is used in several biomedical applications, such as pharmaceutical formulations, cell culture, and tissue engineering [47-50] owing to their biocompatibility, biodegradability, low immunogenicity, water solubility, low cost, and easy modification via chemical-crosslinking other materials. The clinically approved gelatin-based TAs are listed in Table 2.

**Table 2:** List of clinically approved gelatin-based adhesives (modified from ref. [22])

Company name (country name)	Trade name	Components	Clinic applications	Ref.
**Microval (Australia)**	GRE Biological Glue	Gelatin, resorcinol, formaldehyde, glutaraldehyde	Hemostasis, thoracic aortic dissection	[51]
**Terumo Medical Corp.** **(Japan)**	Angio-Seal	Collagen	Hemostasis	[52]
**Life Bond (Spain)**	Life Seal	Gelatin, microbial transglutaminase	Staple-line leakage	[53]

##### Mechanism of adhesion

**Fig. 5:**
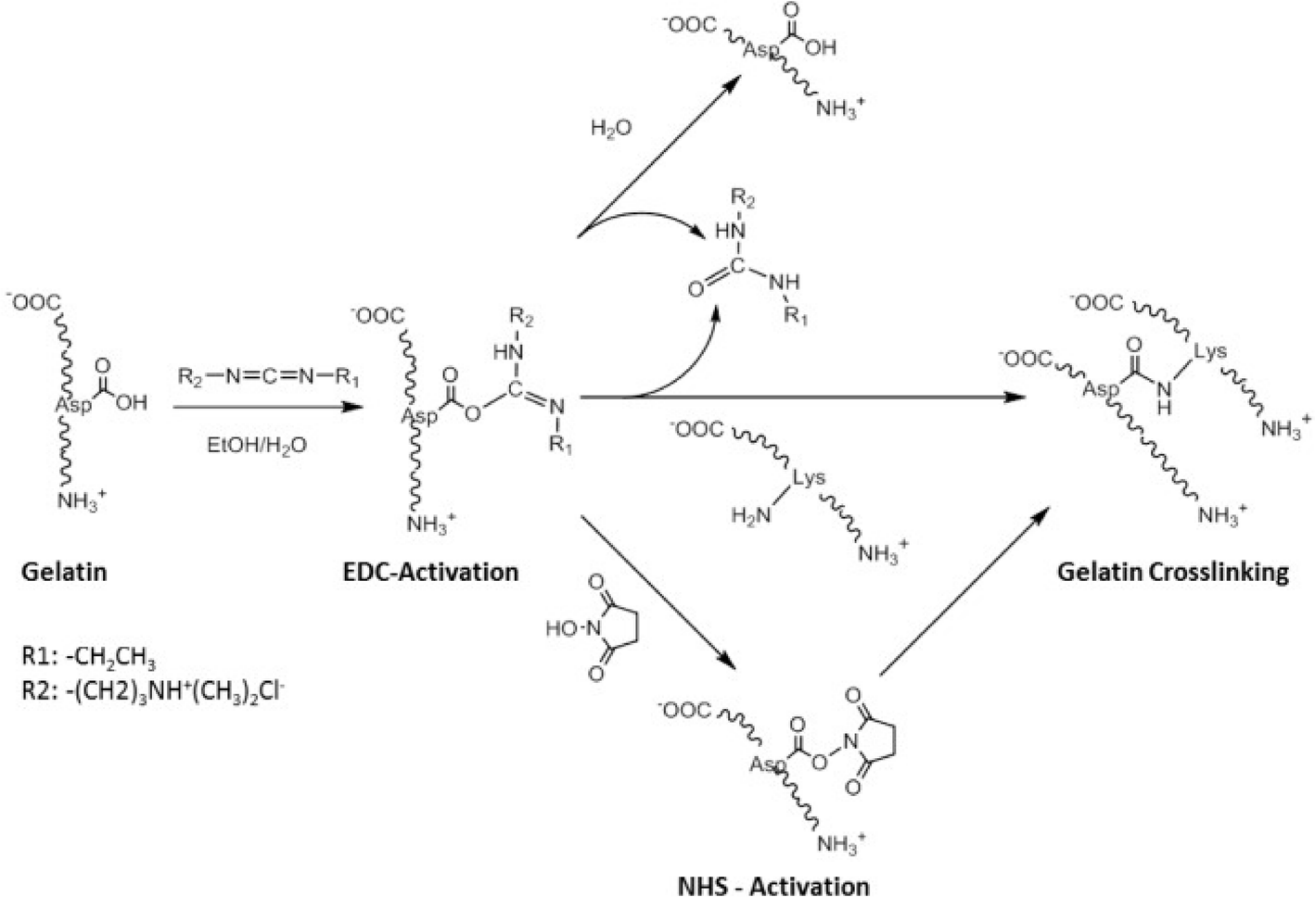
Schematic of the mechanism of the crosslinking reaction between carboxylic acids and lysine, through activation with 1-ethyl-3-(3-dimethyl-aminopropyl)carbodiimide (EDC) and *N*-hydroxysuccinamide (NHS). The amide bond is formed directly between the two amino acids of gelatin with no linker in between. (Adapted from Rose et al. Materials(Basel). 2014;7(4):3106–3135 [58])

Formaldehyde or glutaraldehyde is mixed with gelatin to facilitate the crosslinking of gelatin. Among the crosslinked gelatins, gelatin-resorcinol–formaldehyde/glutaraldehyde (GRFG) is the widely used TA [54]. The two-component solutions are reconstituted for adhesion using gelatin. One component solution is a mixture of gelatin and resorcinol, whereas the other one is a mixture of formaldehyde and glutaraldehyde as crosslinkers. Formaldehyde induces the rapid crosslinking of gelatin, and glutaraldehyde facilitates long-term stability [15]. The two aldehydes react with the amines of gelatin, and formaldehyde is crosslinked with resorcinol when mixed with these two components. Reactions occur within a few min [55]. Additionally, gelatin can be rapidly crosslinked to form a highly elastic TA using the photochemical method via covalent di-tyrosine crosslinking [56]. Gelatin was also crosslinked by employing a biochemical method using microbial transglutaminase via matrix crosslinking between the amines and glutamines using an enzyme [57]. Furthermore, the gelatin can be crosslinked by EDC/NHS activating agents because both compounds activate carboxylic acid residues in the gelatin by nucleophilic attack of free amine groups of lysine on the activated carboxylic acids as shown in Fig. 5 [58].

Recently, mussel-inspired TAs have been attracted because the mussels as the marine animals secrete catechol moiety-rich adhesive proteins and they have strong water-resistant adhesive properties [59]. The catechols like 3,4-dihydroxy phenylalanine (DOPA) play a vital role in the interfacial attachment and enabling them to stick to many different surfaces even in various hydrophilic conditions. Interestingly, Moazami et al., prepared multifunctional mussel-inspired TAs consisting of poly (DOPA-co-acrylate)(PDA), bredigite (BR) NPs, and Fe^3+^ for bone fracture healing during median stenotomy surgery [60] because the PDA has a strong adhesive property in a wet condition and the BR can accelerate the mineralization of calcined tissues with mechanical properties [61]. The TAs exhibited strong adhesion of around 45.9 Mpa to cow skin tissues through irreversible covalent and reversible noncovalent crosslinking depending on the content of BR with an acceleration of in vitro bone-like apatite formation and antibacterial properties although they did not check in vivo.

Fan et al. prepared a mussel-inspired double-crosslinked GE-based TA composed of dopamine-conjugated GE macromer, Fe^3+^ as a rapid crosslinker and genipin as a long-term acting crosslinker for internal medical use [62] because the catechol groups can perform strong wet adhesion on tissue surfaces, and the formation of catechol-Fe^3+^complexation and accompanying rapid curing of genipin-primed covalent crosslinking of gluing GE macromere in one pot through the double-crosslink adhesion mechanism. The new TA exhibited significantly higher wet tissue adhesion ability than commercially available fibrin glue on wet porcine skin and cartilage. Also, the TA showed sound biodegradability and excellent cyto/tissue biocompatibility after subcutaneous implantation of TA in nude mice model although they did not check the TA in vivo.

#### Applications

##### Hemostatic agents

Bleeding is a severe complication of surgery, which may cause perioperative morbidity [63]. The gelatin adhesives crosslinked with formaldehyde and glutaraldehyde were used in vascular surgery [64]; however, the chemical crosslinkers caused tissue toxicity and severe inflammatory activity within a short time [65]. The gelatin TAs, prepared via photopolymerization, were used in gastrointestinal (GI) surgery as a highly elastic tissue sealant for the effective sealing of GI incisions [56]. Additionally, these TAs crosslinked with the microbial transglutaminase were used as the biomimetic sealant in liver surgery with the complete hemostasis of the rat liver in 2.5 min and exhibited substantial adhesive and cohesive strengths [66]. Furthermore, hydrophobically modified gelatin TAs, such as Alaska pollock-derived gelatin mixed with a poly (ethylene glycol)-based four-armed cross-linker [67] or an electrospun gelatin fiber sheet [68] with high interfacial strengths, were used in lung surgeries when ex vivo experiments were performed on extracted rat lungs.

##### Ophthalmic application

**Fig. 6:**
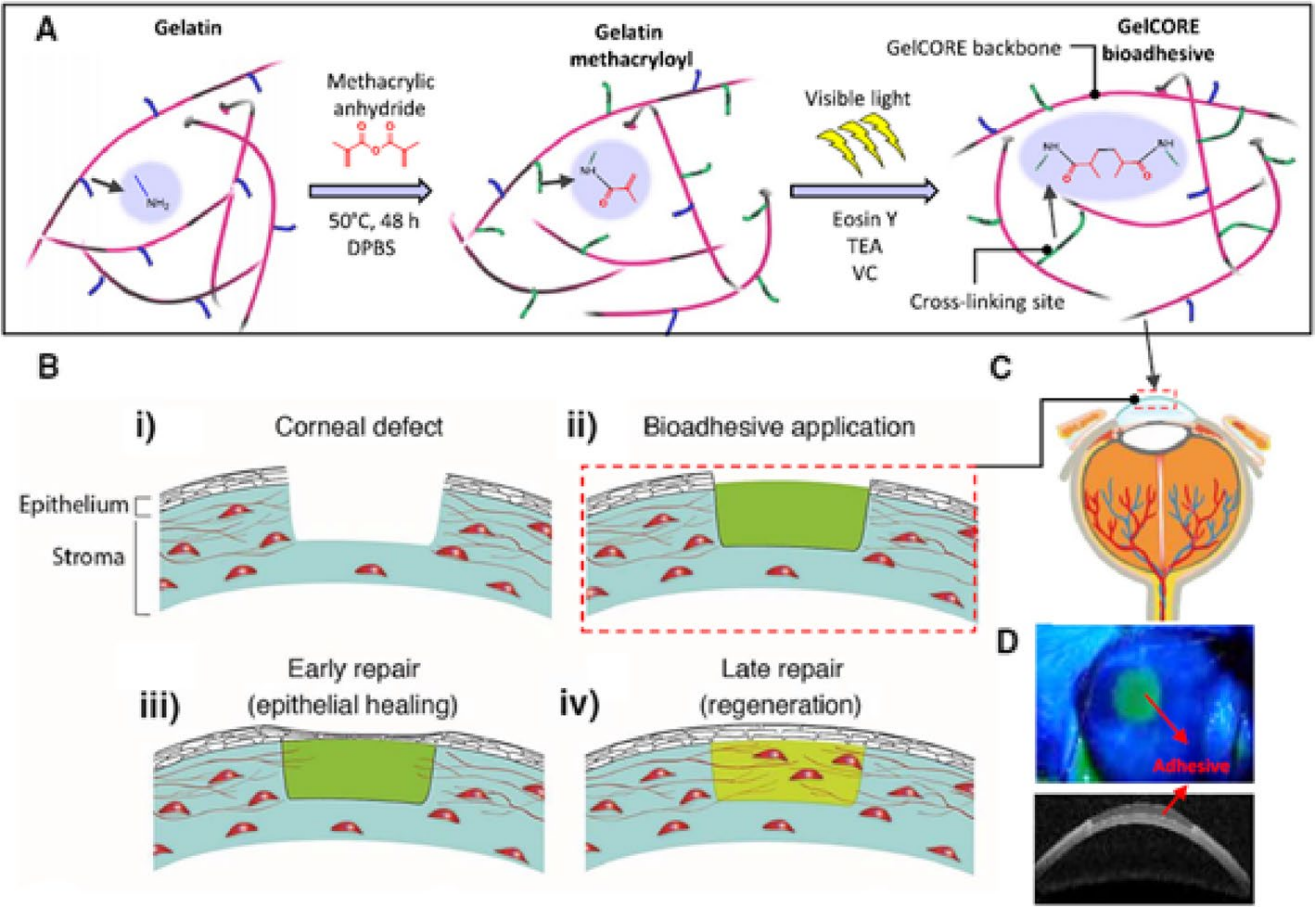
Synthesis, application, and in vitro characterization of GelCORE adhesive hydrogels: **A** Schematic of the chemical reaction for GelCORE formation and photocrosslinking of the prepolymer solution with Eosin Y (photoinitiator), TEA (co-initiator), and VC (co-monomer); **B** Schematic of the application of GelCORE for rapid and long-term repair of corneal injuries, which include: (i) formation of stromal defect, (ii) application of the bioadhesive, (iii) regeneration of the epithelial layer, and (iv) stromal regeneration. **C** Injecting the prepolymer solution into the corneal defect and exposing it to the visible light, which forms: D an adhesive GelCORE hydrogel. (Adapted from Sani et al. Science Advances 2019;5:eaav1281 with permission from AAAS [71])

**Fig. 7:**
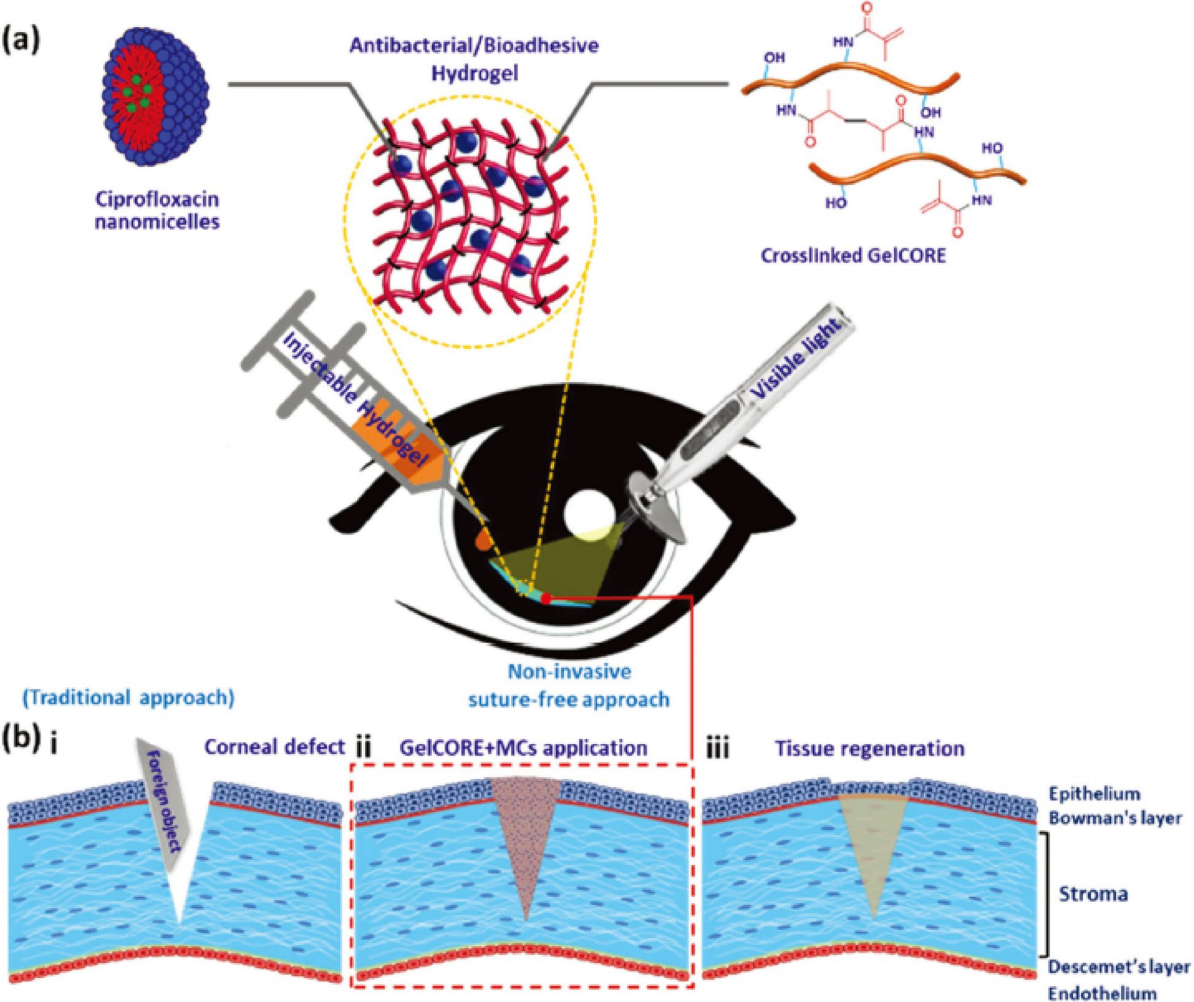
Schematic of non-invasive approach for the treatment of corneal injury with infection: **A** Schematic of the MC-loaded GelCORE adhesive application as a suture-free approach. MC-loaded GelCORE (antibacterial bioadhesive hydrogels) was formed by photocrosslinking the prepolymer solution in the presence of Eosin Y (photoinitiator), TEA (coinitiator), and VC (comonomer). **B** Schematic of the MC-loaded GelCORE application for corneal tissue regeneration after injury with a sharp object, including: (i) formation of a corneal laceration, (ii) application of MC-loaded GelCORE, and (iii) regeneration of the epithelial layer and stromal regeneration. (Adapted from Khalil et al. Biomaterials Science 2020;8:5196–5209 with permission from Royal Society of Chemistry [72])

A high prevalence of ocular trauma is estimated at 3% of all visits to the emergency room [69]. Most of these injuries are vision-threatening because of their occurrence in the cornea. Severe corneal injuries require the use of TAs [70]. Sani et al. [71] prepared a bioadhesive hydrogel methacrylated gelatin via a reaction between gelatin and methacrylic anhydride under visible light for the treatment of corneal injuries. The methacrylated gelatin was photopolymerized via short-term exposure to visible light. The prepared transparent hydrogel firmly adhered to the corneal tissue and exhibited higher tissue adhesion than commercial adhesives. Additionally, this facilitated easy delivery to the cornea for precise bioadhesive curing. Furthermore, these hydrogels effectively sealed corneal defects and induced stromal regeneration and re-epithelialization in a rabbit stromal defect model (Fig. 6) [71]. Khalil et al. [72] synthesized antibacterial bioadhesive hydrogels by loading ciprofloxacin (CPX) into the methacrylated gelatin-based bioadhesive hydrogels, prepared under visible light, for the treatment of corneal injury. The drug-loaded hydrogel exhibited excellent antibacterial activity against *Pseudomonas aeruginosa* and *Staphylococcus aureus* without affecting the adhesive properties of methacrylated gelatin-based bioadhesive hydrogels. In addition, the drug-loaded hydrogel demonstrated a significant decrease in bacterial colony-forming units and high corneal epithelial viability in an ex vivo model of infectious pig corneal injury (Fig. 7) [72]); however, in vivo investigation is required. Sharifi et al. [73] grafted glycidyl methacrylate on the gelatin backbone at significantly low intensities of visible light to prepare a bioadhesive hydrogel, named as glycidyl methacrylated gelatin, with better mechanical properties than methacrylated-based gelatin hydrogels for the treatment of the cornea injury. The prepared hydrogel was modulated to be stretched up to four times of their initial length with high tensile stresses up to 1.95 MPa, which could seal full-penetrating corneal defects of up to 4 mm diameter in ex vivo fresh porcine eyes.

##### Advantages and disadvantages

Gelatin-based TAs offer several advantages, such as biocompatibility, biodegradability, non-inflammatory and non-foreign body reactions, and induction of cell adhesion. However, the molecular weight of gelatin covers a broad range, according to the collagen denaturation method [74]. In addition, the porcine-derived gelatin is gel-like at room temperature because of its high imino acid content; therefore gelatin TAs, which are prepared using porcine-derived gelatin, require heat treatment before being used in surgery [75].

#### Albumin

##### Characteristics

Approximately 50%–60% of the total plasma protein content is the human serum albumin, which is a monomeric single-chain protein secreted by liver cells [76]. Albumin exhibits several biological properties, such as the regulation of the plasma volume and the maintenance of osmotic pressure as well as functions as drug carriers and TAs [77, 78]. The clinically approved albumin adhesives are listed in Table 3.

**Table 3:** List of clinically approved albumin adhesives (modified from ref. [22])

Company name (country name)	Trade name	Components	Clinic applications	Ref.
**CryoLife Inc. (USA)**	BioGlue	Bovine serum albumin and glutaraldehyde	Hemostasis in surgery	[75]
**Neomend Inc. (USA)**	ProGel	Human serum albumin and PEG-NHS ester	Lung parenchyma resection	[79]
**Baxter (USA)**	PreveLeak	Bovine serum albumin and polyaldehyde	Hemostasis in vascular reconstruction	[80]
**Neomend Inc. (USA)**	Tridyne	Human serum albumin and PEG	Hemostasis in aortic surgery	[81]

##### Mechanism of adhesion

**Fig. 8:**
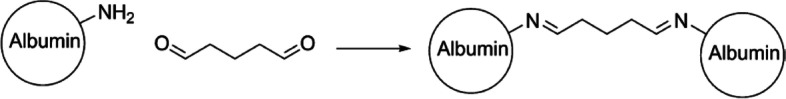
Crosslinking between albumin and glutaraldehyde gives a network. (Bouten et al. Progress in Polymer Science. 2014;39:1375–1405 with permission from Elsevier [82])

The albumin-based adhesive was first synthesized in 1993 via a reaction between the aldehyde groups of glutaraldehyde and amines of albumin as shown in Fig. 8 [82], which was used as a tight mechanical seal and approved by the FDA in 2001 with the registered name BioGlue [83]. The gelation of BioGlue is extremely fast (2 min), and the BioGlue remains in situ for a longer duration than other adhesives; however, it causes an inflammatory response [84]. Furthermore, the bovine origin of the albumin may pose a risk of transmitting infectious agents and allergic reactions [82]. Therefore, polyaldehyde was used as an alternative to aldehyde to react with albumin for reducing the leaching of reactive species, generated from polyaldehyde-treated albumin, with lesser inflammation than that using BioGlue. In another study [79], albumin was rapidly crosslinked with NHS ester-functionalized PEG via a reaction between the negative charges of albumin and NHS esters of PEG through curing for 15–30 s, achieving an adequate strength within 2 min. Albumin adhesive was also prepared via a reaction between an albumin prepolymer, which was obtained through a reaction of albumin and citrate acid via EDC/NHS chemistry, and dopamine [85]. Similarly, an albumin adhesive was prepared via a reaction of albumin with genipin as a natural crosslinker in a water bath at 35℃ for 24 h without further purification [86]. The prepared dark-blue, fluorescent adhesive exhibited a temperature increase owing to the heating-induced curing when irradiated with a near-IR laser.

#### Applications

##### Hemostatic agents

Albumin-based TAs are used to seal pulmonary air leaks [87] and to perform nephron-sparing [88], cardiac [89], nasal septal [90], proximal aortic [81], splenic [91], and inguinal hernia surgeries [92]. However, these TAs cannot control active bleeding; therefore, they can be used to adhere only to a bloodless area. Additionally, BioGlue was reported to cause acute nerve injury, myocardial necrosis [83], and pulmonary embolization [93].

##### Nerve repair Applications

Albumin-based TAs are used for peripheral nerve repair in complete action injuries because sutures cannot heal the injured nerve without leakage of intraneural fluids from the regenerating nerve and often induce detrimental scarring [86]. The albumin TA, crosslinked with genipin using laser tissue welding, exhibited welding of the distal and proximal nerve edges in direct repairs, as well as flexibility and increased adhesion strength [94]; however, it caused inflammatory reactions to the epineurium of the rat sciatic nerves [95].

##### Advantages and disadvantages

Albumin-based TAs are suitable for lung and cardiac repair owing to their excellent shear and tensile strengths and easy availability with fast crosslinking [25]. However, several disadvantages, such as low viscosity, difficult operation, and risks of catastrophic complications and viral infections, have also been reported for these adhesives [25].

### Synthetic polymer-based TAs

#### PCA

##### Characteristics

PCA-based TAs are widely used in medicine, industry, and household usage because they adhere and bind to the target surface within ~ 5–6 s of contact with nucleophiles, such as water and amines, and form a strong film within 60 s [96]. The strong PCA films formed at the tissue surface rapidly degrade into cyanoacetate and formaldehyde without metabolism and elimination, thereby leading to inflammation [97]. The rate of degradation decreases with the increasing length of the alkyl chain. Methyl and ethyl chains of PCAs were first used in medicine, such as abdominal and eye surgeries [82]. Although the cyanoacrylate (CA) monomers cause irritation in the eyes, nose, and throat, the polymerization of CA monomers significantly reduces toxicity [25]. An aeration system is necessary to minimize monomer toxicity. The PCA-based TAs exhibit excellent hemostatic and anti-bacterial properties; therefore, they can be used as microbial barriers [98]. The clinically approved PCA-based TAs are listed in Table 4.

**Table 4:** List of Clinically approved polycyanoacrylate adhesives (modified from ref. [22])

Company name (country name)	Trade name	Components	Clinic applications	Ref.
**B. Braun Medical Inc** **(USA)**	Histoacryl, Histoacryl Blue and Hisoacryl Flexible	Cyanoacrylate derivatives	Closure of topical skin incisions and microbial barrier	[99]
**Ethicon, J & J** **(USA)**	IFAbond	2-Octyl cyanoacrylate	Topical application on skin edges and trauma-induced lacerations	[99]
**Covidien LP** **(UK)**	Indermil	η-Butyl cyanoacrylate	Closure of topical skin incisions in low-skin tension	[99]
**Cordis Neurovascular Inc** **(USA)**	Trufill	η-Butyl cyanoacrylate and ethiodized oil	Embolization of cerebral arteriovenous malformations	[100]
**Ethicon** **(USA)**	Omnes	2-Octyl cyanoacrylate and butyl lactoylcyanoacrylate	Vascular reconstructions	[101]
**Matrix (Italy)**	Glubran2	η-Butyl cyanoacrylate	Surgeries for laparoscopic	[102]
		and methacryloxysulfone	incisions and digestive tract endoscopy	
**Chemence Medical** **(USA)**	Derma + Flex	2-Octyl cyanoacrylate and η-butyl cyanoacrylate	Topical application in skin edges and trauma-induced lacerations	[103]
**LiquiBand Technology** **(UK)**	LiquiBand Exceed	2-Octyl cyanoacrylate	Closure of topical skin incisions and trauma-induced lacerations	[103]
**Adhezion Biomedocal LLC (USA)**	SurgiSeal	2-Octyl cyanoacrylate	Closure of topical skin incisions and trauma-induced lacerations	[104]

##### Mechanism of adhesion

**Fig. 9:**
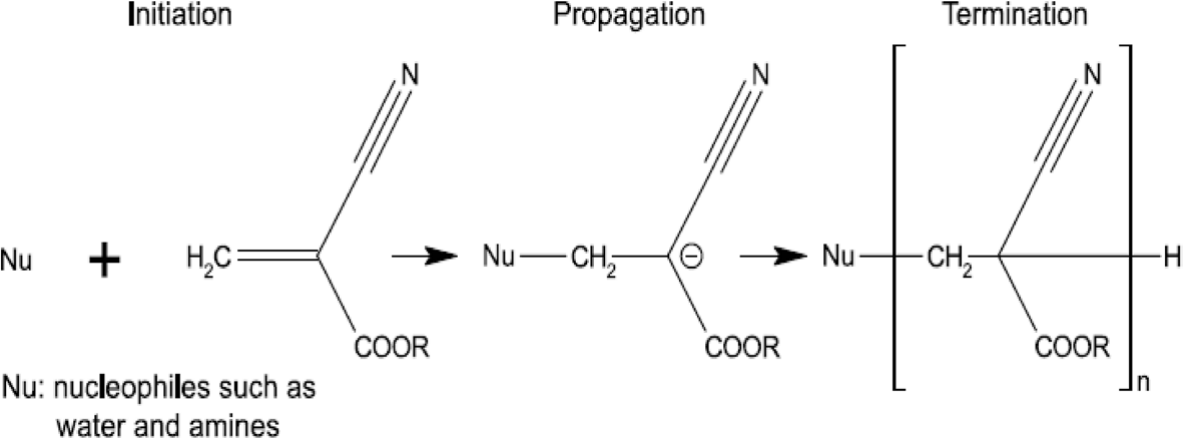
Polymerization process of cyanoacrylates in the presence of weak bases. (Adapted from Klemarczyk et al. Polymer. 2001;42:2837–2848. with permission from Royal Society of Chemistry [106])

The CA monomer comprises a nitrile group and an alkoxy carbonyl group. Both groups are highly electronegative; therefore, the unsaturated carbon double bonds of the acrylate groups undergo Michael addition reactions with nucleophilic compounds, such as water and amines [105]. Subsequently, the produced zwitterion reacts with other accessible monomers in the propagation state (Fig. 9) [106]. During polymerization, covalent crosslinking between the PCAs and amines in the tissue occurs within a few seconds. Finally, the polymeric reaction stops when the monomer accessibility is terminated [107].

#### Applications

##### Hemostatic agents

PCA-based TAs are clinically used as hemostatic agents in treatments related to gastric variceal bleeding [108], laparoscopic fundoplication [109], peptic ulcers [110], ruptured hepatocellular carcinoma [111], nonvariceal upper gastrointestinal bleeding [112], pulmonary vein-esophageal fistula [113], and esophageal variceal ligation-induced ulcer bleeding [114] owing to their excellent ability to control hemorrhages, although a few of them exhibited rebleeding rates for hemorrhages compared to band ligation [115].

##### Plastic surgery

Toriumi et al. [116] prepared a PCA-based TA using 2-octyl CA for clinical applications in wound closure of the face, head, and neck to achieve strong closure and pleasing scars. In addition, PCA-based TAs are used as dressings to cover suture lines and hold back the pinna in the postauricular area without causing any wound infection [117], major skin necrosis, wound dehiscence, or subcutaneous hematoma.

##### Hernia repair

PCA-based TAs are used in hernia surgery to replace traditional sutures to avoid adhesions and uncomfortable parietal nerve trapping. Losi et al. synthesized a PCA-based TA using 2-butyl CA for mesh fixation during hernia repair [118] and demonstrated that the TA was successfully mesh-fixated during hernia repair without any inflammatory reaction. Paajanen et al. [119] used a 2-butyl CA-based PCA TA for mesh fixation in local anesthetic Lichtenstein hernia repair. The mesh fixation without sutures was feasible in Lichtens hernioplasty without compromising the postoperative outcomes. Kukleta et al. [120] synthesized PCA TA using 2-butyl CA for mesh fixation in laparoscopic inguinal hernia repair and demonstrated efficiency and safety with long-term biocompatibility using clinical data. Bellon et al. [121] developed PCA TAs using 2-hexyl and 2-octyl CAs for mesh fixation in hernia repair. Both TAs showed a good mesh fixation with a higher tensile strength than traditional sutures without cell damage responses.

##### Fistula closure

Hosseini et al. [122] synthesized a PCA-based TA using 2-butyl CA to use in pediatric surgery for fistula closure because of tissue fragility and to avoid the major surgery of the patient. The synthesized TA protected the wounds of cloacal exstrophy from colostomy contamination and infection, indicating a promising treatment of fistula. Ortiz-Mogano et al. [123] clinically combined 2-butyl CA-based PCA TA with resolution clips for the endoscopic closure of a rectovaginal fistula. The combined PCA TA/clip improved the endoscopic treatment of rectovaginal fistula because of the rapid solidification of body fluids by PCA-based TA and a scaffold action of the clip as a glue. Barakat et al. [124] used the 2-octyl CA-based PCA TA for topical application to prevent postoperative pancreatic fistula after pancreaticoduodenectomy. A significantly lower rate of postoperative pancreatic fistula was observed in patients who were treated using the PCA-based TA compared to that in the patients who were not treated with it. Leyon et al. [125] clinically used 2-butyl CA-based PCA TA to treat cranial and spinal dural arteriovenous fistulas. Using TA, patients could be successfully treated with complete angiographic exclusion of the fistula in a single round of treatment by achieving venous penetration.

##### Wound closure

Ong et al. [126] clinically compared the closure of abdominal wounds using 2-octyl CA-based PCA TA with that using conventional skin stapling devices. The PCA-based TA demonstrated better cosmetic outcomes and higher patient satisfaction than skin staples. Kumar et al. [127] compared the closure of surgical incisions using silk sutures with that using 2-butyl CA-based PCA TA and demonstrated better epithelialization and lesser inflammatory infiltration with better histological healing using the PCA-based TA than that using silk sutures. El-Gazzar et al. [128] clinically used 2-octyl CA-based PCA TA as an adjunct for wound closure after total knee arthroplasty and suggested that wound drainage was lower in the PCA-based TA group than that in the control group. Buchweitz et al. [129] compared the cosmetic outcome of the skin adhesive, prepared by combining 2-octyl and n-2-butyl CA, with that of transcutaneous sutures in laparoscopic port-site wounds and demonstrated that the closure of laparoscopic port-site wounds and cosmetic outcome of the prepared skin adhesive were similar to the transcutaneous sutures. Teah et al. [130] combined the 2-octyl and n-2-butyl CA to prepare a PCA-based TA and compared its aesthetic performance with subcuticular sutures obtained using PGA in thyroidectomy wound closure. No statistical differences were observed between these two samples in terms of aesthetic performance, median score, and observers’ satisfaction score.

##### Advantages and disadvantages

PCA-based TAs offer several advantages, such as rapid and painless application, no requirement for complicated surgery and complex dressings, the prevention of microorganisms from entering the wounds, low dehiscence rates, and good tolerance and comfort with high patient satisfaction [97]. In contrast, several disadvantages, such as the limited strength in the presence of water or blood, low tensile strength, allergic concerns in patients, adverse reactions to asthma and deep burn wounds, and potential toxicity, have also been reported for PCA-based TAs [131].

#### Poly (ethylene glycol) (PEG)

##### Characteristics

PEG-based TAs have received attention because the PEG is widely used as a biomaterial in drug delivery systems and tissue engineering owing to its hydrophilic and biocompatible properties, easy chemical modification and functionalization, non-immunogenicity, and water solubility [132], even though PEG is not biodegradable. The clinically approved PEG-based TAs are listed in Table 5.

**Table 5:** List of clinically approved PEG-based adhesives (modified from ref. 22)

Company name (country name)	Trade name	Components	Clinic applications	Ref.
Focal Inc. (USA)	FocalSeal	PEG-co-poly(lactic acid) diacrylate and PEG-co-poly(trimethylene carbonate)diacrylate	Closure of visceral pleural air leaks in pulmonary resection	[133]
Covidien (UK)	DuraSeal	PEG NHS ester and trilysine amine	Dural repair of durotomy in the spine for watertight closure	[134]
Baxter Bio Science Inc (USA)	CoSeal	PEG NHS ester and PEG thiol	Hemostasis in vascular surgery	[135]
Ocular Theapeutix Inc (USA)	Resure	PEG and trilysine acetate	Use for clear corneal incisions	[136]
HyperBranch Medical Technology (USA)	OcuSeal	PEG aldehyde and cysteine-terminated lysine dendron	Protective barrier for corneal, conjunctiva, and sclera surfaces	[137]
Covidien (UK)	SprayGel	PEG NHS ester and PEG amine	Gynecological and colorectal surgeries for adhesion barrier	[138]
Stryker Corp.(USA)	Adherus	PEG NHS ester and hyperbranched poly(ethylene imine)	Dural repair of cerebrospinal fluid leaks for watertight closure	[139]

##### Mechanism of adhesion

**Fig. 10:**
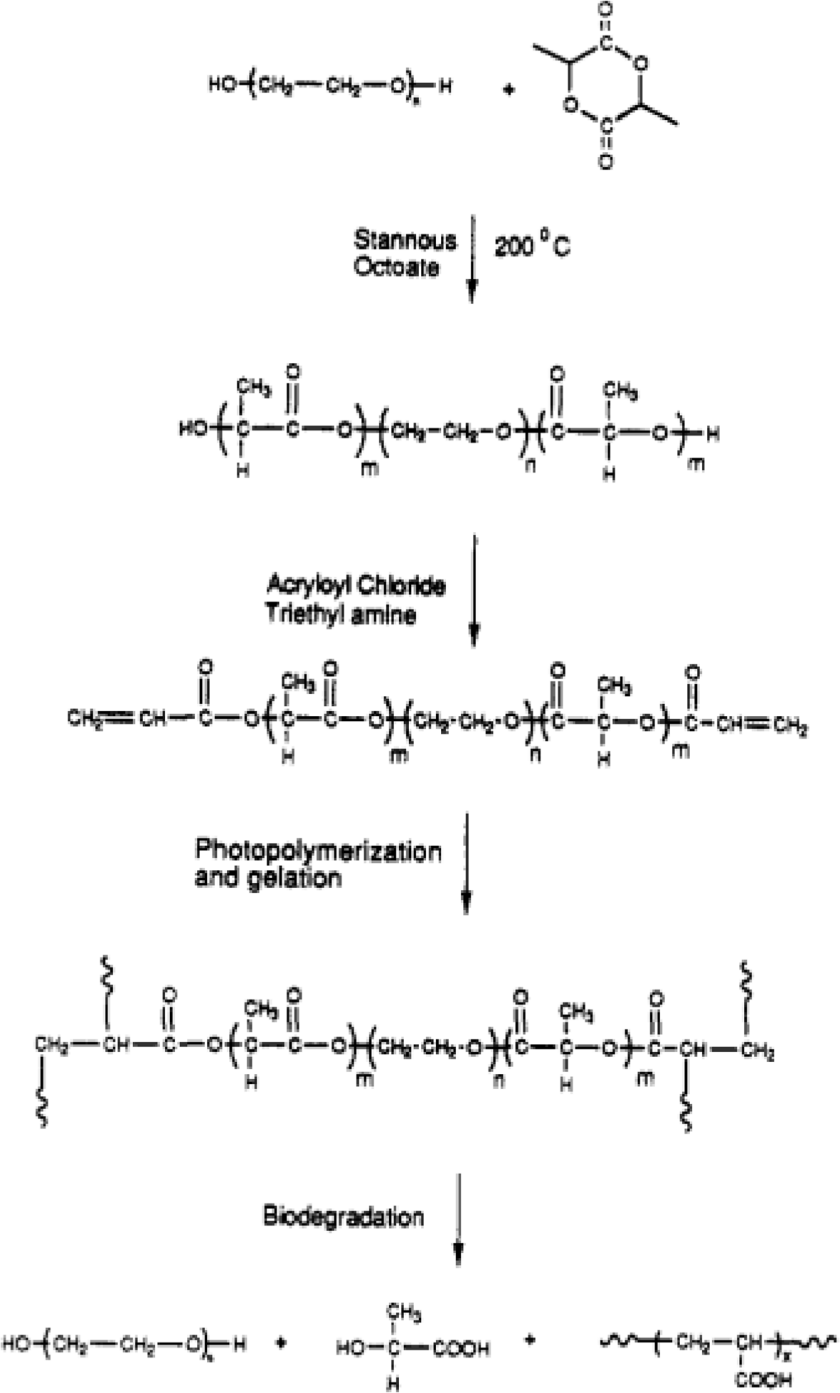
Reaction scheme for the synthesis of polymerizable PEG-co-poly(a-hydroxy acid) di- and tetraacrylates and hydrogels, as well as their degradation. (Adapted from Sawhney et al. Bioerodible Hydrogels Based on Photopolymerized Poly(Ethylene Glycol)-Co-Poly (α-Hydroxy Acid) Diacrylate Macromers. Macromolecules 1993;26:581 − 587. with permission from American Chemical Society [141])

PEG-based TA was first developed using macromers containing a PEG as a central block, extended with poly (α-hydroxy acids), such as poly (lactic acid) (PLA) or poly (glycolic acid) (PGA), and terminated with acrylate groups as shown in Fig. 10 [140]. The macromers were rapidly photopolymerized with a photoinitiator in direct contact with tissues without local toxicity. Subsequently, the hydrogel-type adhesives adhered to the tissues because of the formation of interpenetrating networks with the tissues. The first clinically approved PEG-based TAs comprised two macromers: PLA-PEG-PLA triblock and poly(trimethylene carbonate) (PTMC)-PEG-PTMC triblock copolymers, which were terminated with acrylate groups [82]. For tissue adhesion, a solution of PLA-PEG-PLA diacrylate was first deposited onto the tissue surface as a primer layer, and a solution of PTMC-PEG-PTMC diacrylate was added onto the primer layer. Subsequently, the two macromers were polymerized using eosin Y as a photoinitiator. The prepared hydrogel-type adhesives were used to seal air leaks during the lung surgery. Another PEG-based TA comprised two components: a four-armed PEG end-functionalized with NHS esters and trilysine as a tetra amine crosslinker [141]. When applied to tissues, the NHS esters of PEG reacted with the amines of the crosslinker and formed a hydrogel adhesive in the tissues within a few min. Similarly, the use of a PEG-based TA comprising starPEG functionalized with amines and starPEG with NHS esters resulted in the formation of a hydrogel adhesive on tissues with a postoperative adhesion barrier, which remained on tissues for up to a week after colorectal surgery [138]. In addition, PEG-based TA, comprising starPEG functionalized with thiols as the crosslinker and starPEG with esters formed a hydrogel adhesive owing to the production of thioesters via a reaction of thiols with NHS esters [135]. The thioesters then underwent trans-amidation with amines in the tissues. A hydrogel adhesive was formed within 30 s using a PEG-based TA, comprising PEG end-functionalized with ester aldehydes and a cysteine-terminated lysine dendron with four cysteines via a reaction of 1,2-thiolamine group of cysteine with the aldehydes of PEG in a stepwise sequence [137]. Other PEG-based TAs were prepared via the template polymerization of acrylic acid (AA) in the presence of PEG [142] or PEG macromers (PEGM) [143]. The mucoadhesive force of the PEG/poly (acrylic acid)(PAA) or PEGM/PAA was stronger than the mucoadhesive force of a commercial adhesive Carbopol 971PNF, owing to the formation of polymer complexes between PEG and PAA via hydrogen bonding; however, their adhesive properties at the tissues were not investigated.

Shimony et al. prepared liquid copolymers as a new type of TAs [144] because the prepolymers hardens upon mixing of PEG_4_-PLGA-NHS and PEG_4_-NH_2_ to yield an elastic biodegradable sealant. The TAs exhibited longer persistence time with stronger mechanical properties than fibrin glue in vitro although the mechanical properties and crosslinking time are dependent on the ratio of the two prepolymers. Also, The TAs showed a capsule formation at only subcutaneous injection sites to the flank of rats whereas the commercial Dermaband showed prominent ECM necrosis and pyknotic nuclei, indication of a promising TA solution for wound closure.

Yang’s group prepared injectable mussel-inspired PEG-based TAs by the reaction of citric acid, PEG and dopamine via a one-step polycondensation reaction to make prepolymer and, then crosslinking of the prepolymer in the presence of sodium periodate as an oxidizing agent for wound closure [145] because the citric acid is used to not only form degradable polyesters with PEG but also to provide pendant reactive carboxyl groups to conjugate dopamine. The TAs showed 2.5–8 folds stronger wet tissue adhesion on porcine small intestine than clinically used fibrin glue. Also, the TAs stopped bleeding instantly and closed wounds created on the back of rats with controlled degradability and excellent cyto/tissue compatibility whereas the fibrin glue was impossible due to the weak wet tissue adhesion. Also, they prepared another magnesium oxide (MgO)-crosslinked mussel-inspired PEG-PPG-PEG-based TAs by the reaction of citric acid, PEG-PPG-PEG diol and dopamine by a one-pot polycondensation reaction [146] because the PEG-PPG-PEG instead of PEG can reduce swelling ratio due to the hydrophobic property than PEG and the MgO can act as both crosslinking initiators and composite fillers for enhancing the adhesion and biocompatibility. The TAs crosslinked by MgO with sodium periodate exhibited high adhesion strength of 125 kPa on porcine intestine submucosa ex vivo, eightfold that of fibrin glue with high mechanical strength of about 4.5 Mpa. Also the TAs showed good wound closure efficacy on rat skin incisions with excellent in vitro and in vivo biocompatibility due to the added MgO.

#### Applications

##### Hemostatic applications

PEG-based TA prepared using PEG ester and trilysine was applied in spinal surgery [147] with effective wound closure; however, they exhibited significant adhesive swelling. Other PEG-based TAs prepared using PEG with glutaryl-succinimidyl ester and thiol terminal groups were applied in vascular surgeries; however, the hydrolysis of ester and thioester linkages resulted in the degradation of the adhesives [148]. In addition, photocurable PEG-based TA, which was prepared using styrene-derivatized gelatin and PEGDA under visible light, was used for artery surgery in Wistar rats [149].

##### Ocular applications

PEG-based TAs, prepared using NHS-terminated four-arm succinimidyl-glycolate PEG and amine-functionalized four-arm PEG, were applied to central corneal incisions in an ex vivo rabbit model [150]. The crosslinked hydrogels were formed within a few s. They exhibited stability for up to six weeks and could be successfully sealed with a high leakage pressure tolerance. Another PEG-based TA, prepared using NHS-terminated four-arm PEG and trilysine amine, was used in cataract surgery and single-plane incision [70]. Stable hydrogels were formed within 30 s after mixing and exhibited higher efficiency in averting fluid egress, faster healing, and better lubrication after cataract surgery than sutures in addition to providing improved comfort to the patient. Moreover, the PEG-based TAs were used in conjunction with flap lifting and scraping to prevent the recurrence of epithelial ingrowth in patients undergoing laser-assisted keratomileusis [151]. The ocular performances were good owing to the non-recrudescence of interface epithelium; however, epithelial ingrowth was observed in other uneventful lasers. Furthermore, Hoshi et al. [152] synthesized a clear, flexible, and firmly adherent hydrogel under xenon irradiation for closing retinol breaks in porcine and rabbit eyes using PEG-based TA, which was prepared using (PEG-co-PLADA) and (PEG-co-PTMC) DA. The adhesive effectively sealed retinal breaks without detachment from the retina, and no inflammatory reaction or toxicity was observed in the eyes for 28 d.

##### Advantages and disadvantages

PEG-based adhesives offer advantages, such as biocompatibility and easy control over chemical modification for their functionalities. However, weak mechanical properties, low cohesive strength with brittleness, high swelling properties owing to their hydrophilic nature, and multiple preparation steps for adhesive application are the major disadvantages of these adhesives [82].

#### Polyurethane (PU)

##### Characteristics

PUs have received increasing attention in biomedical applications, such as catheters, stents, blood oxygenators, cardiac valves, dressings, drug delivery carriers, tissue engineering [153], and tissue adhesives, owing to their mechanical flexibility, good wettability, biocompatibility, tailorable foam, and high tear strength [154]. PUs can be synthesized via nucleophilic addition polymerization between the isocyanate and polyol compounds [155]. The physicochemical properties of PUs can be adjusted owing to the availability of several functional groups with flexible or rigid chains [156].

##### Mechanism of adhesion

**Fig. 11:**
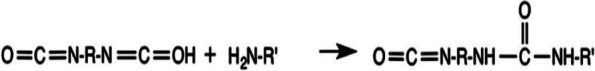
Tissue adhesion mechanism of urethane-based adhesive. H_2_N-R`represents tissue amines that react with isocyanate groups through urea bond formation. (Adapted from Scognamiglio et al. Applied Biomaterials, 2016; 1048(3):626–639, with permission from Wiley Online Library[1])

PU-based adhesives generally comprise isocyanate-terminated prepolymers and water molecules, when contacted with the biological state [157]. These prepolymers covalently react with tissue via urea linkages between the isocyanate and amine groups of tissue (Fig. 11) [1].

**Fig. 12:**
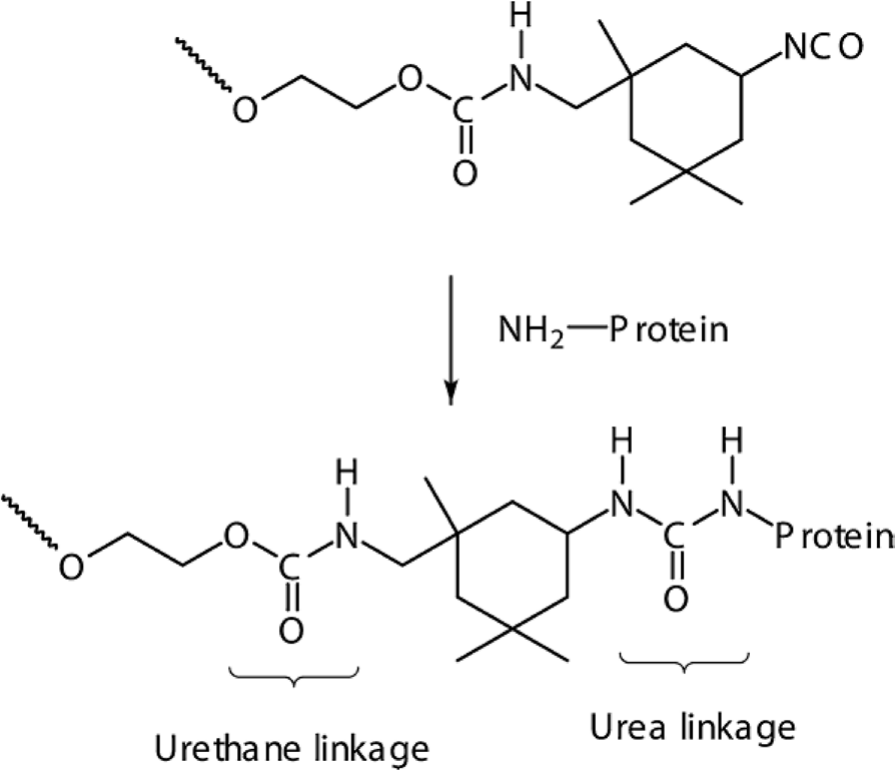
Reaction between a prepolymer and the amino groups of a protein resulting in a urea linkage. (Adapted from Ferreira et al. J Mater Sci: Mater Med 2008;19:111–120. with permission from Springer [158])

PU-based adhesives were developed as a biodegradable urethane prepolymer.via a reaction between polycaprolactone (PCL) diol and isophorone diisocyanate or hexamethylene diisocyanate [158]. The obtained prepolymer reacted with the amino groups of living tissues via urea linkage (Fig. 12), which caused thrombosis when it came in contact with blood [158]. Furthermore, the PCL diol was modified with 2-isocyanate ethyl methacrylate (IEMA) to prepare a prepolymer that was photo crosslinked under UV irradiation [159], which caused hemolysis when it came into contact with blood. Phaneuf et al. [160] developed a PU-based adhesive comprising polyether-based PU with carboxylic acid groups to create anchor sites in tissues; however, this adhesive did not exhibit sealant properties. The PU prepolymer synthesized by a reaction of lysine-diisocyanate and lysine-triisocyanate with diols and polyols via urethane linkages was developed by Cohera Medical Inc., USA [27]. The remaining isocyanates in the prepolymer crosslinked with themselves via urea linkages in presence of water as a weak base. The tissue adhesion occurred because of the reaction of isocyanates in the prepolymer with tissue amines within 25 min. Furthermore, the same company developed a PU prepolymer via a reaction of lysine-based isocyanate with a PEG prepolymer and triethoxysilane [161]. The tissue adhesion occurred rapidly owing to the reaction of isocyanates in the prepolymer and tissue amines in the presence of moisture without any safety risks. The clinically approved PU-based TAs are listed in Table 6.

**Table 6:** List of clinically approved PU-based adhesives (modified from ref. [22])

Company name (country name)	Trade name	Components	Clinic applications	Ref.
**Conera Medical Inc. (USA)**	TissuGlu	Lysine, isocyanate, diol (or polyol)	Application for abdominoplasty surgery	[162]
**Conera Medical Inc. (USA)**	Sylys	Lysine, isocyanate, PEG, triethoxysilane	Adjunctive closure during gastrointestinal procedures	[161]
**Adhesys Medical (USA)**	MAR-CUTIS(Flix)	Unknown	Topical application for skin wound Closure	[109]

#### Applications

##### Hemostatic applications

PU-based TAs are used in renal [163], endocrinology [164], pancreatic duct occlusion [165], and colorectal surgeries [160]. Particularly, the PU-based TAs, prepared by reacting lysine isocyanate and diols, are used in abdominoplasty surgeries in dogs and humans without fluid accumulation [166].

##### Bone fixation

PU-based TAs were prepared via the reaction of 4,4-diphenylmethane diisocyanate (MDI) with poly (tetramethylene ether glycol) (PTMEG) and subsequent addition of 2,2-bis (hydroxymethyl)-propionic acid. The prepared adhesive was used for bone fixation [160] because vascular grafts are slightly permeable to blood and cause leakage of blood into the body [82]. However, in vivo studies were not performed. Lei et al. [167] prepared a foam-like PU-based TA by reacting Polycin D-290, which is a castor oil derivative of polyol, with polyisocyanate prepolymer and β-tricalcium phosphate in the presence of water and a catalyst for promoting bone tissue growth. The adhesion strength of the adhesive to the porcine rib bone in an ex vivo experiment was two times higher than that of the clinically approved poly(methyl methacrylate) (PMMA) bone cement. Additionally, the porous structure of the PU-based TA facilitated the growth of cells and bone tissues in a rabbit model. Blanguer et al. [168] developed PU-based TAs via a reaction of poly (trimethylene carbonate) (PTMC) diol with butane diisocyanate (BDI) to treat annulus fibrosus (AF) in intervertebral disk degeneration. The adhesive properties of these TAs were assessed in a lap-shear tensile test, indicating a significantly stronger adhesion of the prepared adhesives to the caudal AF tissue than that of the fibrin glue. However, in vivo experiments were not performed.

Recently, Balcioglu et al., prepared fast curing multifunctional gentamicin-loaded TAs based on UV-curable PU acrylate as a prepolymer synthesized via two stages for sternal closure [169] because the TAs can overcome the disadvantages of use of wire cerclage after sternal closure such as its rigidity and strength. The resulting TAs exhibited the highest adhesion strength of 4322 kPa on glass slides with high biocompatibility and antibacterial properties because silk sericin, PEG, and dopamine as biocompatible OH agents, and 4,4’-methylenebis (cyclohexyl isocyanate) as a less cytotoxic NCO agent were used to prepare the prepolymers although the adhesion strength was dependent on the molecular weight of PEG. Also, the TAs exhibited no visible inflammation when the TAs were applied to bond the bones and cured with UV for 5 min after opening the sternum of rats whereas the PCA as a control group showed very high inflammation, indication of possibility of sternal fixation with a tissue healing.

##### Advantages and disadvantages

PU-based TAs offer several advantages, such as biocompatibility, biodegradability, reaction with amino groups of proteins present in the tissues, excellent thermal stability at physiological temperature, and non-hemolytic behavior [82]. In contrast, these adhesives may cause potential toxicity because of degradation products, containing several components of isocyanates, polyols, catalysts, chain extenders, and crosslinkers during PU preparation. Furthermore, optimizing these bioadhesives according to different types of tissue is difficult.

## Conclusion and perspectives

The development of novel TAs has received considerable attention in TA-based applications owing to the reduction in surgery time, less pain, leakage prevention, and the absence of removal procedure requirements [1]. Sutures are widely used to close and seal wounds because of their simple and rapid application procedure. However, the pain and discomfort caused by invasive techniques have compelled the development of sutureless methods. Fibrin-based TAs are used in different surgical procedures owing to their hemostatic properties and orthopedic applications; however, poor adhesion under wet conditions and the risk of virus transmission restrict their applicability. Gelatin-based TAs are used as hemostatic agents in ophthalmic applications owing to their biocompatibility, biodegradability, and non-inflammatory reactions. However, cytotoxicity is the primary concern when aldehyde-containing materials are used for crosslinking with gelatin. Albumin-based TAs are used as hemostatic agents in nerve repair applications owing to their excellent shear and tensile strengths, easy availability, and fast crosslinking; however, the low viscosity and risk of catastrophic complications and viral infections are the associated disadvantages. PCA-based TAs are primarily used in plastic surgery, wound closure, fistula closure, and hernia repair as hemostatic agents because of the rapid and painless application procedures, non-complicated surgery, the prevention of microorganisms from entering the wounds, low dehiscence rates, and good comfort with high patient satisfaction. However, they exhibit limited strength in water, low tensile strength, allergic concerns, and potential cytotoxicity. PEG-based TAs are used as hemostatic agents in ocular applications owing to their biocompatibility and easy control over chemical modification for the functionalities. However, these adhesives exhibit weak mechanical properties, low cohesive strengths, and high swelling properties and require multiple preparation steps for their applications. PU-based TAs are used for hemostatic agents and bone fixation because of their biocompatibility with possible biodegradability, thermal stability, and non-hemolytic behavior; however, the potential toxicity of degradation products and difficulty in optimizing bioadhesive properties depending on the tissue types are the primary concerns associated with these TAs.

Currently, a significant gap exists between the research efforts devoted to TAs and the number of available products. To bridge this gap, designing TAs with a deep understanding of the target tissue surface characteristics and environment is essential. Furthermore, monitoring the long-term performance of the used TAs, including chemical and physical properties after their usage, as well as the tissue responses to TAs should be implemented. Moreover, understanding the regulatory and development pathways for applying TA technologies in clinical trials should be considered. Additionally, a research collaboration among material scientists, molecular biologists, and clinicians is required. Furthermore, the development of next-generation TAs comprising biomimetic materials, such as those mimicking the functions of mussels, slugs, geckos, and octopuses, is speculated to provide sufficient adhesion strength depending on diverse tissue settings and multifunctional properties.

## Data Availability

All data is available upon request to the corresponding author.

## Abbreviations

TAs: Tissue adhesives
PCA: Polycyanoacrylate
PEG: Poly(ethylene glycol)
PU: Polyurethane
PSAs: Pressure-sensitive adhesives
BM-hMSCs: Bone marrow-derived human mesenchymal stem cells
THA: Total hip arthroplasty
IV: Intravenous
TXA: Tranexamic acid
THR: Total hip replacement;
GRFG: Gelatin-resorcinol–formaldehyde/glutaraldehyde
GI: Gastrointestinal
CPX: Ciprofloxacin
CA: Cyanoacrylate
PLA: Poly(lactic acid)
PGA: Poly(glycolic acid)
PTMC: Poly(trimethylene carbonate)
PEGM: PEG macromer
PAA: Poly(acrylic acid)
PCL: Polycaprolactone
IEMA: 2-Isocyanate ethyl methacrylate
PTMEG: Poly(tetramethylene ether glycol)
PMMA: Poly(methyl methacrylate)
PTMC: Poly (trimethylene carbonate)
BDI: Butane diisocyanate
AF: Annulus fibrosus
